# Reconstruction of the Cytokine Signaling in Lysosomal Storage Diseases by Literature Mining and Network Analysis

**DOI:** 10.3389/fcell.2021.703489

**Published:** 2021-08-20

**Authors:** Silvia Parolo, Danilo Tomasoni, Pranami Bora, Alan Ramponi, Chanchala Kaddi, Karim Azer, Enrico Domenici, Susana Neves-Zaph, Rosario Lombardo

**Affiliations:** ^1^Fondazione the Microsoft Research-University of Trento Centre for Computational and Systems Biology, Rovereto, Italy; ^2^Data and Data Science – Translational Disease Modeling, Sanofi, Bridgewater, NJ, United States; ^3^Department of Cellular, Computational and Integrative Biology (CIBIO), University of Trento, Trento, Italy

**Keywords:** cytokine, text-mining, natural language processing, systems biology, lysosomal storage diseases, Gaucher, Fabry, ASMD

## Abstract

Lysosomal storage diseases (LSDs) are characterized by the abnormal accumulation of substrates in tissues due to the deficiency of lysosomal proteins. Among the numerous clinical manifestations, chronic inflammation has been consistently reported for several LSDs. However, the molecular mechanisms involved in the inflammatory response are still not completely understood. In this study, we performed text-mining and systems biology analyses to investigate the inflammatory signals in three LSDs characterized by sphingolipid accumulation: Gaucher disease, Acid Sphingomyelinase Deficiency (ASMD), and Fabry Disease. We first identified the cytokines linked to the LSDs, and then built on the extracted knowledge to investigate the inflammatory signals. We found numerous transcription factors that are putative regulators of cytokine expression in a cell-specific context, such as the signaling axes controlled by STAT2, JUN, and NR4A2 as candidate regulators of the monocyte Gaucher disease cytokine network. Overall, our results suggest the presence of a complex inflammatory signaling in LSDs involving many cellular and molecular players that could be further investigated as putative targets of anti-inflammatory therapies.

## Introduction

Lysosomal storage diseases (LSDs) are a group of rare metabolic disorders in which a defect in the gene encoding a lysosomal protein causes the accumulation of substrates inside the lysosome (Platt et al., 2018). This ultimately leads to numerous clinical manifestations. Among them, several LSDs have been associated with abnormalities of the immune system and chronic inflammation (Bosch and Kielian, 2015; Pandey et al., 2017, 2018; Rigante et al., 2017).

Despite the growing body of evidence supporting a connection between LSDs and inflammation, the existing knowledge is distributed across numerous scientific publications, and a unifying knowledgebase is still missing. Text-mining is increasingly used to extract knowledge from unstructured text of scientific articles (Huang and Lu, 2016; Przybyla et al., 2016; Westergaard et al., 2018; Lee et al., 2020). Successful examples of text-mining in the biomedical field include extractions of gene-disease associations (Piñero et al., 2015; Zhou and Fu, 2018), protein–protein interactions (Saik et al., 2016; Szklarczyk et al., 2019), drug discovery (Azer et al., 2019; Zheng et al., 2019; Hansson et al., 2020) and clinical trial design (Michelini et al., 2018). Text-mining has also been applied to the study of the immune system. Recently, a cell-cytokine network was built by PubMed mining (Kveler et al., 2018), and specific conditions such as the immune response to psychological stress (Priyadarshini and Aich, 2012) or the immune-related adverse events of immuno-oncology drugs (Yu et al., 2020) were investigated by mining of the literature. The application of text-mining to rare diseases is a less explored area; however, it could provide an important contribution to better understand the underlying biological processes that drive clinical manifestations and to develop new treatments (Boycott and Ardigó, 2018; Sakate et al., 2018; Shen et al., 2018; Roessler et al., 2021).

In this study, we developed an integrative analysis based on text-mining and network analysis to study the cytokines involved in three related LSDs: Gaucher Disease (GD), Acid Sphingomyelinase Deficiency (ASMD), and Fabry Disease (FD).

Gaucher Disease is caused by a deficient lysosomal β-glucosidase activity, which results in the accumulation of glucosylceramide (GL1) in macrophages, leading to hepatosplenomegaly, anemia, skeletal lesions, and, in some cases, neurological manifestations. Three main forms of GD are usually distinguished: type 1 (GD1) is the chronic non-neurological form, type 2 (GD2) is the acute neurological form that leads to premature death in early childhood and type 3 (GD3) is the chronic neurological form also associated with damage to peripheral tissues (Sidransky, 2004; Stirnemann et al., 2017). Currently, pharmacological treatments are available for the non-neuronopathic forms of GD and include both enzyme replacement therapies (ERT) and substrate reduction therapies (SRT) (Weinreb et al., 2013; Peterschmitt et al., 2018). ASMD is caused by mutations in the gene encoding the lysosomal enzyme acid sphingomyelinase, that converts sphingomyelin to ceramide in lysosomes. Historically, ASMD has been referred to as Niemann-Pick Disease. While an infantile neurovisceral type of ASMD (ASMD type A previously known as NPD A) has an extremely severe course involving the central nervous system (CNS), and usually a premature death in early childhood, ASMD type B (NPD B) typically displays visceral and pulmonary involvement without CNS involvement and a more heterogeneous time course (Schuchman and Desnick, 2017). Olipudase alfa, a recombinant human acid sphingomyelinase is currently in clinical trials for AMSD treatment (Thurberg et al., 2020). FD is caused by mutations in the gene encoding the enzyme alpha galactosidase A that leads to globotriaosylceramide accumulation mainly in endothelial cells, kidney cells, and cardiomyocytes. In agreement with this, the most relevant clinical manifestations of FD are quite different from those of GD and ASMD and they include renal failure, cardiac and cerebrovascular disease (Wanner et al., 2018). ERT therapy is available for FD as well (Azevedo et al., 2021).

Independently of the disease-specific therapies, the treatment of chronic inflammation is emerging as a potential adjuvant therapy for LSD patients (Platt, 2018), and a comprehensive analysis of the inflammatory signaling involved in LSDs can help to understand the dysregulated pathways. With that goal, computational mining of literature performed in this study resulted with the identification of a list of cytokines associated with GD, FD, and ASMD, which were then used as seed for a systems biology workflow providing insight into the inflammatory processes associated to LSDs.

## Materials and Methods

### Text-Mining Pipeline

Over 31 million abstracts from PubMed and 6.1 million full texts from PubMed Central were harmonized and indexed on a Solr^1^ instance along with the clinical trial descriptions from ClinicalTrials.org. Documents underwent an automatic annotation of genes, proteins, diseases, species and chemicals leveraging state-of-the-art Machine Learning (ML) methods DNorm (Leaman et al., 2013), GNorm (Wei et al., 2015), Huner (Weber et al., 2020), TaggerOne (Leaman and Lu, 2016), MutationFinder (Caporaso et al., 2007). The tagged entities were organized and curated along with paper’s keywords and MeSH terms to identify the relevant search terms. Word2Vec (Mikolov et al., 2013; Pyysalo et al., 2013) was also used to identify lists of mentions appearing in contexts statistically similar to the ones of the input keywords. A variety of data-driven search terms were identified in this way, including some frequent mistypings (e.g., “neimann-pick”) and other non-standard mentions. Starting from the simple “Gaucher disease,” “Fabry disease,” and “ASMD” queries, we picked relevant search terms from the unbiased information coming from ML data-driven suggestions of annotations. Some exclusions were also easily identified from the structured results, leading to the following three queries used to identify the relevant literature corpora (on the 31st of December 2020):

Title, abstract, keyword, mesh: (“acid beta-glucosidase deficiency” OR gaucher OR “gba deficiency” OR “glucocerebrosidase deficiency” OR “glucosylceramide beta-glucosidase deficiency”).
Title, abstract, keyword, mesh: (fabry OR “Alpha-galactosidase deficiency” OR “alpha-galactosidase A deficiency” OR “GLA deficiency” OR “angiokeratoma corporis diffusum”).
AND NOT title, abstract, keyword, mesh: (“Fabry-Pérot” OR “Fabry-Perot” OR “Pérot-Fabry” OR “Perot-Fabry”).
[Title, abstract, keyword, mesh: (asmd OR “ASM deficiency” OR “Acid Sphingomyelinase deficiency” OR “neimann pick” OR “smpd1 deficiency” OR “smpd1 mutation” OR “neimann-pick” OR “neimann-pick”)].
AND NOT keyword: [“asmd, absolute standardized mean difference” OR “absolute standardized mean difference” OR “adaptive steered molecular dynamics (asmd)” OR “adaptive steered molecular dynamics”].

The same methods described above were used also for the concepts to be mined in the text. The list of concepts corresponding to disease synonyms was defined by querying the following databases and ontologies: OMIM^2^, orphanet^3^, ICD-10^4^, MeSH^5^, disease ontology^6^. Alternative names of the mutated gene/protein were identified from NCBI gene^7^, UniProt^8^, MedlinePlus^9^. Synonyms of the accumulated metabolites and the deacylated forms (lyso-species) were instead retrieved from PubChem^10^ and ChEBI^11^ databases. Specifically, for GD we searched synonyms of glucosylceramide and glucosylsphingosine (lyso-GL1), for FD we searched synonyms of globotriaosylceramide and globotriaosylsphingosine (lyso-Gb3), and for ASMD we searched synonyms of sphingomyelin and lyso-sphingomyelin (lyso-SM). Moreover, we included additional keywords from scientific articles identified in PubMed, from machine-learning annotation of texts and by the expanding the seed terms in the target corpora. The dictionary of cytokines was defined by leveraging the cytokine registry from the ImmPort (Bhattacharya et al., 2018) and the CytReg (Santoso et al., 2020) databases.

Disease synonyms, metabolites, cytokines and genes were identified by the entity recognition task powered by *ad hoc* linguistic variant-detection (Ramponi et al., 2020). Paragraphs where such mentions occurred were not analyzed for co-mention, given that we were not looking at summarization statistics which are known to suffer from poor precision associations in the absence of intensive human screening. The text was rather analyzed using a hi-precision method that proved highly reliable on five different benchmark corpora (Ramponi et al., 2020). The method identified syntactic relational structures among the entities and extracted linguistic associations between cytokines and disease-related concepts. The result is a structured format defining effector, affected and a verbal association among the two, therefore allowing for further systems analysis. The results were filtered to retain only sentences mentioning at least one disease-cytokine association. Sentences from clinical trial records and the method section of the full text articles were excluded due to their low information content. We also excluded from subsequent analyses all cytokine names that could not be mapped unambiguously to a gene symbol.

### Evaluation of Cell-Type Expression Specificity of the Cytokine

Gene expression information for eight blood cell types: eosinophils, basophils, neutrophils, T-cells, B-cells, monocytes, NK-cells, and dendritic cells was downloaded from Human Protein Atlas (HPA) (Uhlén et al., 2015)^12^. We analyzed the data from the consensus dataset in Blood Atlas, and we considered as expressed all genes with an HPA normalized expression value >1, and “cell-specific” all genes classified by HPA as cell-type enriched or group enriched.

### Construction of the Cytokine Gene Regulatory Networks

The transcription factor (TF)–cytokine gene regulatory network (GRN) was taken from CytReg database (Santoso et al., 2020). Disease specific, immune cell TF-cytokine networks were created by first selecting human interactions and filtering the original network to retain only the cytokines identified by text-mining, and then filtering the resulting network to keep only the genes expressed in the cell type of interest (HPA normalized expression value >1). The co-expression between the TF-cytokine pairs of the GD monocyte GRN network was tested using the blood monocyte expression data downloaded from HPA Blood Atlas. For each gene, we computed the average monocyte expression at sample level (average of classical monocyte, intermediate monocyte, and non-classical monocyte) and we performed the correlation test using the Pearson method. The cytokine GRN network of GD monocyte was visualized using Cytoscape, version 3.7.1^13^.

### Ligand-Receptor Analysis

Ligand-receptor (LR) pairs were downloaded from CellTalkDB (Shao et al., 2020). Pathway enrichment analysis was performed using the enrichPathway function from the r package ReactomePA, which uses the hypergeometric test to evaluate whether specific Reactome pathways are enriched in a gene list (Yu and He, 2016). As background genes we used all the cytokines present in the dictionary used for text-mining and their receptors, as reported in CellTalkDB. Cytokines not present in CellTalkDB were not included in the background gene list. The minGSSize was set equal to 5. To control for multiple testing, we used the Benjamini-Hochberg correction. To build the disease-immune cell networks, we identified the cytokine receptors from CellTalkDB. We then assessed the expression of the genes encoding cytokine and receptors produced by the immune cells using the HPA data described above, and we removed non-expressed genes from the networks. The cell-cell interactions were scored based on the number of LR pairs connecting them. By analyzing the frequency distribution of the number of LR pairs connecting the cells, we selected the cell-cell interactions with a number of LR pairs above the 75th percentile and we created the network. The igraph package for R was used to create and plot the networks^14^.

### Enrichment Analysis of Cell-Specific Cytokines

We defined the lists cell-specific cytokine-sets by selecting for each cell the genes encoding cytokines that HPA Blood Atlas reports as “cell-type enriched” or “group enriched.” The gene-set collection of cell-specific cytokines was built using the loadGSC function of the Bioconductor package piano. The enrichment of GD cytokines in monocyte-specific cytokines was tested using the Fisher’s exact test implemented in the runGSAhyper function of the Bioconductor package piano. The analysis was performed using all the cytokines present in the cytokine dictionary used for the text-mining analysis corresponding to a human gene symbol as universe.

### Search of Transcriptomic and Proteomic Datasets

To identify relevant transcriptomic datasets, NCBI Gene Expression Omnibus (GEO)^15^ and EBI ArrayExpress^16^ were queried on 12th July 2021. The searches were performed using the following keywords: ‘‘gaucher’’ and ‘‘fabry.’’ GD proteomic datasets were identified by searching Omics DI^17^ using the keywork “gaucher” and NCBI PubMed using the query “proteomic[TIAB] AND gaucher[TIAB]” (search performed on 20th July 2021). The results were manually curated to exclude non-relevant datasets.

## Results

### Text-Mining Analysis Identifies a List of Cytokines Associated With LSDs in the Literature

**FIGURE 1:**
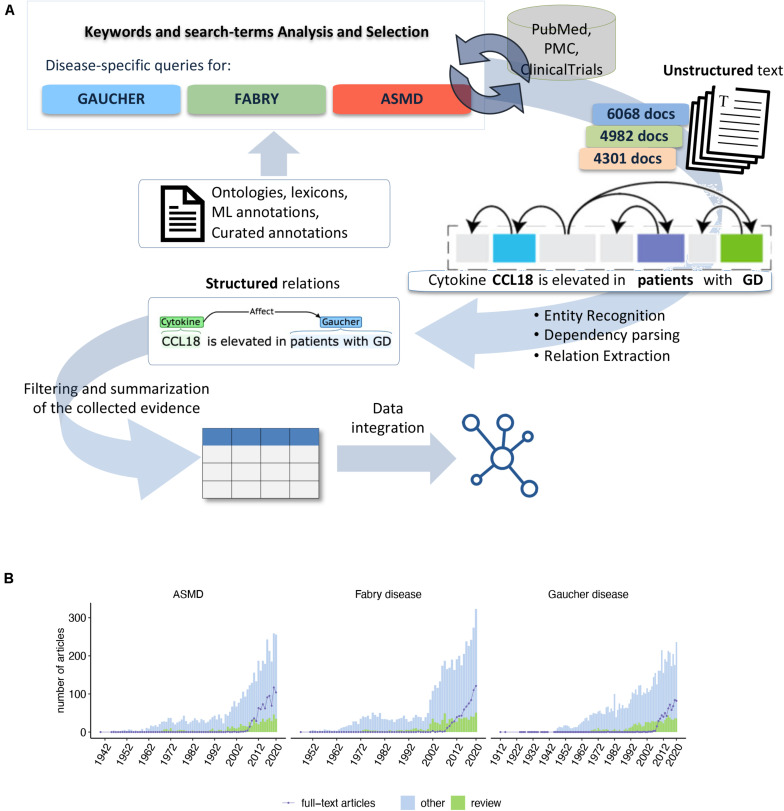
**(A)** Overview of the text-mining pipeline: Ontologies, DBs, and ML annotations were used to select specific queries and search terms on PubMed, PMC for the three diseases. Over 15,000 documents were retrieved and processed with our NLP-based analytics platform. The structured knowledge derived from the linguistic relationship extraction was normalized, analyzed, and integrated with biological databases to derive a systems outlook of the inflammatory signal. **(B)** Summary of the disease-specific scientific literature we analyzed to identify the cytokines. For each disease is shown the number of articles per year of publication. The number of review articles is highlighted in green. The violet line shows the number of full-text articles available for text-mining.

To identify the cytokines associated with GD, FD, and ASMD, we devised a text-mining pipeline that uses Natural Language Processing (NLP) techniques to process all PubMed and PubMed Central (PMC) documents to identify sentences describing a relationship between disease-related concepts and at least one cytokine (Figure 1A). First, we collected a corpus of scientific publications related to the three diseases. The available documents are both PubMed citations and full-text articles. Indeed, due to copyright restrictions, in some cases the complete article was not available for text-mining, and only the citation (including title, abstract, and the MeSH terms) could be automatically parsed. For GD, we identified 6,068 articles published from 1912 to 2020, 917 of which were review articles. Among all the available documents, 693 were full-text articles, and the others were citations. For FD, we retrieved a set of 4,982 documents published between 1947 and 2020, out of which 809 were review articles. The full text was available for 730 documents. For ASMD, we identified 4,301 articles mentioning ASMD or Niemann-Pick (NP) disease in the text having a range of publication dates from 1940 to 2020. In this case, the number of identified review articles was 648 and the number of full-text documents available for the text-mining analysis was 853 (Figure 1B). The NP disease mentions were retrieved as described in the methods and further classified in type A, B, C, D, E, F, and generic NP, allowing to filter only for NP type A and B in the relations.

In addition to the search of scientific articles, we also compiled a catalog of publicly available transcriptomic datasets related to the three diseases. By querying NCBI Gene Expression Omnibus and ArrayExpress, we identified 17 GD transcriptomic studies. Twelve studies are derived from GD mouse models, four from GD human tissues/cells and one from an experiment in fruit flies. For FD, four studies were identified, three derived from mouse models and one from human organoids. For ASMD, no publicly available transcriptomic study was identified (Supplementary Table 1). We also searched for published GD proteomic studies by querying Omics DI (see text footnote 17) and NCBI PubMed and we identified four published proteomics studies (Supplementary Table 1). For FD, a recent review already compiled a comprehensive list of proteomic studies (Rossi et al., 2021) and we could not identify any additional study. We did not find any ASMD-related proteomic study.

**FIGURE 2:**
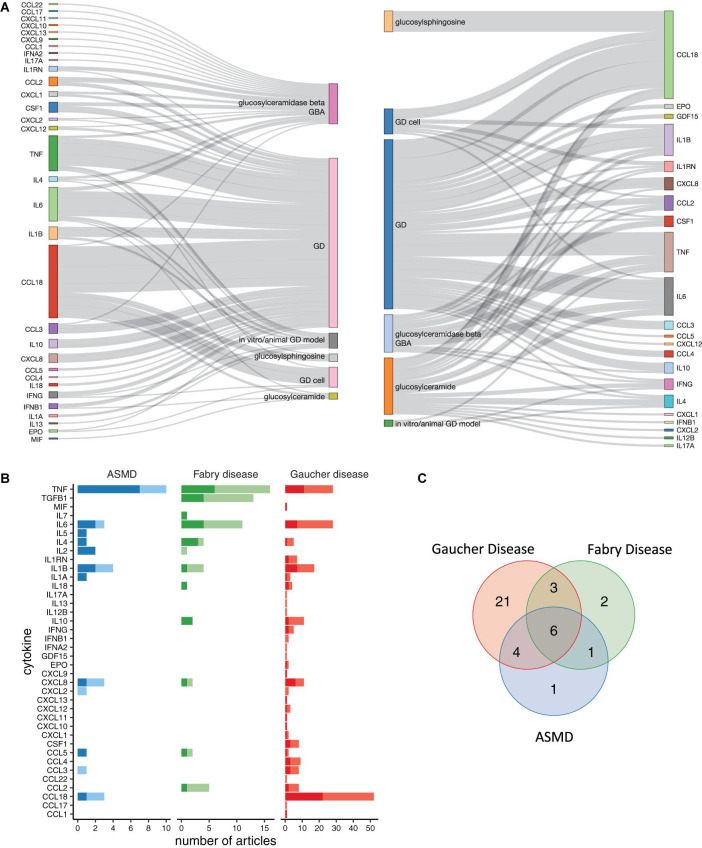
Cytokines identified by the text-mining pipeline. **(A)** Linguistic structure of the mined text, shown as a bi-partite information flow, in the GD literature. The chart summarizes the directional associations between cytokines and the disease-related terms. The relations derived from sentences in which the cytokine is the subject are shown on the left, instead those in which the cytokine is more of an object are shown on the right. **(B)** The chart shows the number of papers mentioning each cytokine in association with the disease. Darker colors indicate that at least one sentence mentioning cytokine-disease association is present in the title or abstract of the article, suggesting that the identified association is probably a key finding. Lighter colors indicate the cytokine-disease associations derived from other sections of the article. **(C)** Venn diagram of the lists of cytokines obtained for the three diseases.

The retrieved scientific articles were analyzed to identify relevant sentences following the approach detailed in section “Materials and Methods” and summarized in Figure 1A. Briefly, the entire set of documents related to the disease of interest was computationally processed to identify sentences with a linguistic relationship between a disease-related concept (disease, mutated enzyme, accumulated metabolites) and at least one cytokine. In this work, we focused on a list of cytokines identified starting from the cytokine registry made available by the ImmPort project (Bhattacharya et al., 2018) and from the CytReg database (Santoso et al., 2020). Growth factors and hormones without a primary role in the immune system and the cytokine receptors were not included in the text-mining search. For GD, we identified 280 relevant sentences from 102 articles (Supplementary Table 2). These sentences mention 44 GD-related cytokines, out of which 34 could be assigned unambiguously to a human gene symbol. A visual summary of the disease-cytokine associations present in the GD literature is shown in Figure 2A. This chart shows the directed relations between the cytokines and the disease-related concepts automatically identified by the text-mining pipeline. The chemokine CCL18 is the most cited cytokine in GD literature, being mentioned together with a disease term in 114 sentences from 52 articles, followed by TNF with 59 sentences from 28 articles (Figure 2B). For FD, the list of cytokines was obtained from 163 sentences in 47 articles (Supplementary Table 3 and Supplementary Figure 1). These sentences report 16 cytokines, and for 12 of them we could find the corresponding gene symbol (Figure 2A). For ASMD, we found 15 cytokines in 72 sentences from 24 articles (Supplementary Table 4 and Supplementary Figure 2), and we identified the corresponding gene symbol for 12 cytokines (Figure 2A). For both FD and ASMD, the most cited cytokine is TNF. It is also worth noting that six cytokines, namely CCL5, CXCL8, IL1B, IL4, IL6, and TNF, are shared among the three lists, suggesting a shared inflammatory signal among the diseases (Figure 2C).

The sentences were manually annotated for mentions of clinical studies, animal models, tissues, and cells (Supplementary Tables 2–4). Overall, 66 sentences from GD results report findings from clinical studies, 17 sentences findings from studies performed on animal models, 38 sentences findings from *in vitro* studies, 6 sentences mixed findings, and 153 sentences do not specify the type of study. Most of the sentences indicating the tissue where the cytokine has been measured report blood findings, with 55 sentences mentioning serum, plasma, or a generic mention to blood/circulation. When we checked the type of disease, we identified 33 GD sentences that are specific for one type of disease, while the others do not specify any GD type.

Most of the FD-related sentences refer to *in vitro* studies (52 out of 163 sentences) and in particular they mention cell cultures of podocytes (11 sentences), a kidney epithelial cell-type particularly affected by globotriaosylceramide accumulation (Waldek and Feriozzi, 2014). *In vitro* studies are also the most common ones in ASMD sentences, with 26 sentences. In this case, most of the studies mentioned by the sentences refer to experiments performed on fibroblasts (22 sentences).

### Immune Cell-Type Specificity of the Identified Cytokines

**FIGURE 3:**
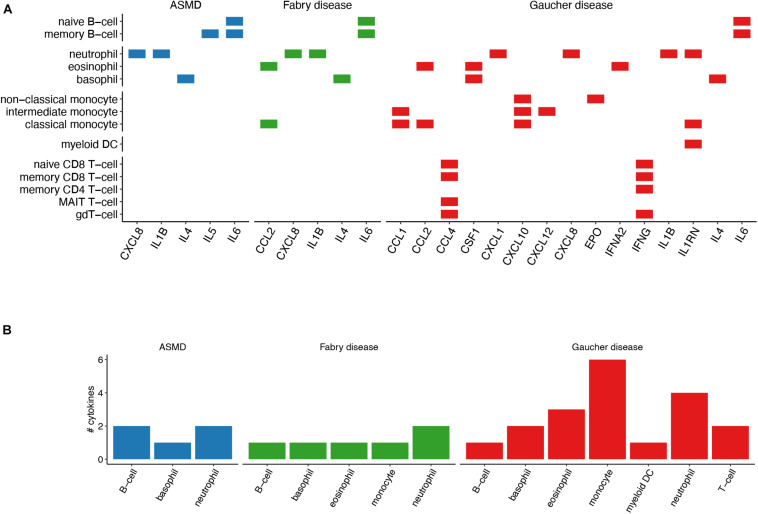
Immune-cell specificity of the identified cytokines. **(A)** The chart shows the cytokines that are reported as cell-specific (cell-type enriched or group enriched) by the HPA Blood Atlas. **(B)** Number of cell-specific cytokines per cell-type. Compared with the analysis in panel **(A)**, B-cells, T-cells and monocytes have been grouped in one cell type.

Since cytokines are molecular mediators of cell communication, mainly among immune cells, we set out to investigate the immune-cell specificity of the cytokines identified by the text-mining pipeline. According to the consensus dataset from the HPA Blood Atlas (Uhlén et al., 2015), 15 literature-derived GD cytokines are cell-specific (cell type enriched or cell group enriched) and six of them, namely CCL1, CCL2, CXCL10, CXCL12, IL1RN, and erythropoietin (EPO), are monocyte-specific (Figures 3A,B). When tested in a overrepresentation analysis, the monocyte-specific cytokine-set resulted significantly enriched in GD cytokines (Fisher’s exact test *p*-value = 0.04). The other cell-specific cytokine-sets were not tested for their enrichment in GD cytokines due to the small number of overlapping genes (less than five genes). It is worth noting that CCL18, the cytokine with the highest number of citations in GD literature, was “not detected” in the HPA Blood Atlas dataset, thus we could not evaluate its cell specificity. We identified cell-specific cytokines also for ASMD and FD. However, in these cases we only found one or two cytokines overlapping the cell-specific cytokine-sets (Figures 3A,B) and thus we could not test the significance of the overrepresentation for any cell-type.

### Construction of a GD-Cytokine Gene Regulatory Network

**FIGURE 4:**
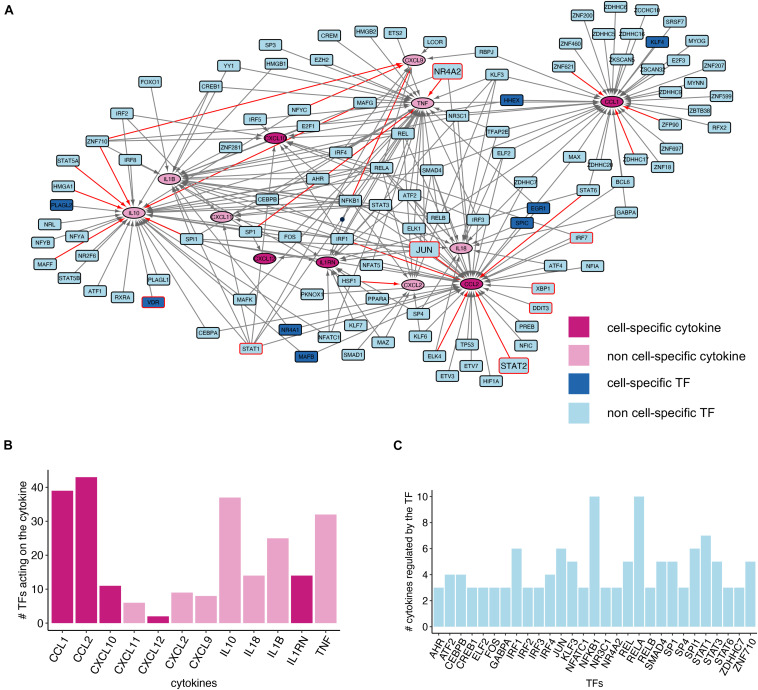
Cytokine gene regulatory network of GD monocytes. **(A)** Network plot showing the TF-cytokine interactions. Only genes expressed in monocytes according to the HPA Blood Atlas data are reported. The nodes with a red border were identified in GD literature and the red edges indicate a significant correlation between TFs and cytokines. The big nodes (NR4A2, JUN, and STAT2) correspond to TFs identified in the GD literature with monocyte gene expression significantly correlated with a cytokine in the network. **(B)** Bar chart showing the number of incoming edges for all the cytokines in the chart, corresponding to the number of putative TFs regulating cytokine gene expression. **(C)** Bar chart showing the number of outgoing edges for the TF nodes with more than two degrees which corresponds to the number of cytokine genes regulated by each TF.

Next, we decided to investigate the gene regulatory network (GRN) behind the expression of cytokine genes identified by text-mining. We focused on the regulation of GD cytokine gene expression in monocytes since this is the cell type with the highest number of cell-specific cytokines (Figure 3B). We built the cytokine GRNs by identifying the TFs regulating the expression of the text-mining derived GD cytokines leveraging the information provided by CytReg, a recently published database of TFs-cytokine interactions (Santoso et al., 2020) which we filtered according to the monocyte gene expression reported in the Blood Atlas data (Figure 4).

Overall, we can observe that numerous TFs contribute to the regulation of cytokine gene expression. Among the identified TFs, there are well-known regulators of genes involved in the immune expression, such as members of the NF-κB (REL, RELA, RELB, and NFKB1) and different members of the interferon-regulatory factor (IRF) proteins family (Figure 4C). Moreover, we noticed the presence of the nuclear receptor NR4A2, a TF encoded by a gene harboring genetic variants that have been associated with familial Parkinson’s disease susceptibility (Le et al., 2003). Similarly, NR3C1 has been associated with epigenetic deregulations in Parkinson’s disease (Fernández-Santiago et al., 2015). Since GD patients are at higher risk of developing Parkinson’s disease (Behl et al., 2021), this finding deserves further investigation.

To investigate the TFs present in the cytokine GRN of GD monocytes, we used TFs as seeds of the text-mining analysis aiming at the identification of TFs reported within the context of GD. This analysis identified seven TFs, namely DDIT3, JUN, IRF7, NR4A2, STAT1, STAT2, VDR, and XBP1 (Supplementary Table 5). DDIT3 and XBP1 are TFs regulating *CCL2* expression in the GRN network and are both involved in the Unfolded Protein Response (UPR), a mechanism activated by the endoplasmic reticulum (ER) to cope with stress conditions. The text-mining analysis identified contradictory findings related to the UPR induction in GD. Indeed, we found both articles reporting UPR activation in GD and PD patients with mutations in *GBA* gene and articles showing lack of evidence (Farfel-Becker et al., 2009; Gegg et al., 2012; Maor et al., 2013; Braunstein et al., 2018; Do et al., 2019; Ivanova et al., 2019). The master regulator of type I interferon signaling IRF7, which in our network regulates *CCL2* and *CXCL10* gene expression (Figure 4), was reported by two studies as elevated in the neurological forms of GD (Vitner et al., 2016; Melamed et al., 2020). The phosphorylated form of STAT2, a regulator of *CCL2* expression in the network in Figure 4, was also up-regulated in the brain of a GD mouse model (Vitner et al., 2016). On the other hand, INFG-induced STAT1 activation was shown to be inhibited in Gaucher cells (Batta et al., 2018). STAT1 in our network regulates several cytokine genes: *IL10*, *IL1B*, *CCL2*, *TNF*, *CXCL10*, *CXCL11*, and *CXCL9*. Our analysis also pointed out a study that investigated *NR4A2* expression in dopaminergic neurons obtained from GD iPSC. This study showed a decrease, albeit not significant, of *NR4A2* expression in these cells (Awad et al., 2017). Moreover, text-mining identified a study indicating that the expression of glucocerebrosidase gene is affected by JUN (Moran et al., 1997), a TF that in our network regulates *IL10*, *IL1B*, *CCL2*, *TNF*, and *CXCL12*. Finally, we identified several studies reporting polymorphisms in the VDR gene possibly associated with GD phenotypes (Vlieger et al., 2002; Greenwood et al., 2010a, b; Lieblich et al., 2011; Zhang et al., 2012; Mistry et al., 2013; Gervas-Arruga et al., 2015; Zimmermann et al., 2018; Kałużna et al., 2019).

We also investigated the correlation pattern of the identified TF-cytokine pairs by leveraging the monocyte gene expression data from the HPA Blood Atlas. In total, we identified 20 TF-cytokine gene pairs with a significant correlation (Supplementary Table 6). Among them, STAT2-CCL2, JUN-CCL2, and NR4A2-TNF involve TFs already described in the GD literature.

### Construction of a Cytokine-Driven Immune Cell-Communication Network

**FIGURE 5:**
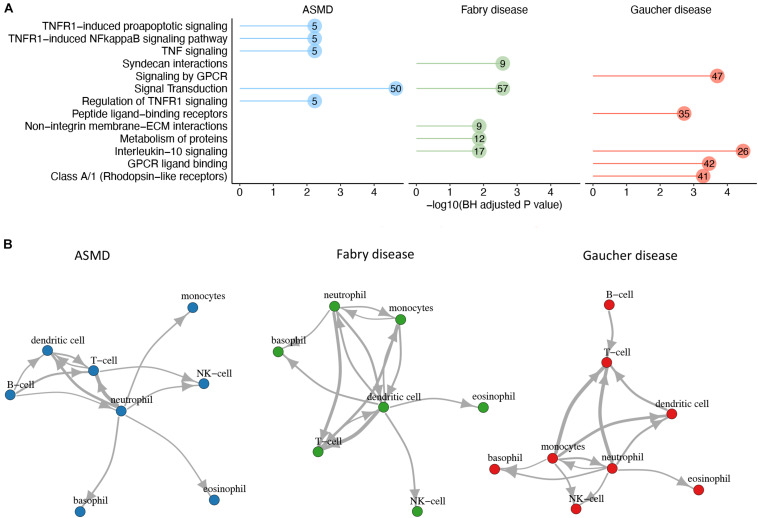
Cytokine-receptor analysis to build cell-cell networks. **(A)** Functional enrichment analysis. For each disease, the top 5 Reactome pathways enriched in disease cytokines and their receptors are shown. The numbers within the dots indicate the number of cytokines/receptors belonging to the pathway. **(B)** For each disease, the top cell-cell interactions involving the literature-derived cytokines and their receptors are shown. The cell-cell interactions were scored based on the number of CR pairs involved, and those above the 75th percentile are shown in the network chart. The thickness of the edge is proportional to the number of interacting CR pairs that in ASMD network ranges from 20 to 34, in FD network from 28 to 44 and in GD from 33 to 56.

Cytokines are molecular effectors that mediate the communication between cells, mainly of the immune system. In this study, we explored the cytokine signaling by leveraging publicly available data on cytokine-receptor interactions. We first identified the receptors of the text-mining derived cytokines from CellTalkDB, a recently published, curated database of ligand-receptor LR pairs (Shao et al., 2020). We identified 180 cytokine-receptor (CR) pairs for GD, 80 for ASMD and 95 for FD. Pathway analysis of the GD cytokines and their receptors identified “interleukin 10 signaling” as the most enriched pathway, with 26 genes. This pathway is also among the top significantly enriched pathways when considering the genes derived from the list of FD cytokines and their corresponding receptors. For ASMD, instead, the most enriched pathways are related to TNF signaling, with five cytokines and receptors (Figure 5A). Having identified the cytokine receptors, we assessed the immune cell expression of the genes encoding the cytokines and their receptors using the gene expression data from the HPA Blood Atlas, and we reconstructed a putative cell-cell interaction network based on the number of CR pairs linking two cells (Figure 5B). In the GD network, the intercellular interaction supported by the highest number of CR pairs is the one going from monocytes (producing the cytokine) to T cells (expressing the receptor), with 58 CR interactions.

In the FD network, the interaction dendritic cells - > T cells is the one with the highest number of CR pairs with 44 CR interactions, while in the ASMD network the cell-cell communication supported by the highest number of CR pairs is between neutrophils and T cells, with 34 interactions.

## Discussion

In this study, we investigated the inflammatory signaling characterizing GD, FD, and ASMD. Our integrative approach starts from literature computational mining to identify the cytokines associated with the three diseases, and then combines the literature findings with other data sources through a systems biology framework. Compared with previous efforts to identify immune cells and molecular mediators by text-mining (Kveler et al., 2018), in this study we focused on three specific LSDs. This allowed us to set up a tailored approach that reflects the characteristics of the LSDs of interest. For example, we could evaluate the association between the specific disease genes (mutated genes) or accumulated metabolites and cytokines. Indeed, the primary driver of the LSD phenotype is the accumulation of lipids that cause cellular, tissue and organ dysfunctions that are frequently coupled with chronic inflammation. The causes of the observed chronic inflammation are still not fully understood. For example, in GD and ASMD, these undegraded lipids mainly accumulate in macrophages (Pandey and Grabowski, 2013), instead in FD the vasculature is particularly affected (Bodary et al., 2007). The accumulation of glucosylceramide in GD macrophages causes their activation, disrupts autophagy and starts a cascade of inflammatory events that worsen the disease itself (Simonaro, 2016). To take into account these aspects in the text-mining analysis, we set up a search for cytokines described in association with accumulating lipids and their deacylated forms. These results were merged with those obtained by searching disease-cytokine and mutated enzyme-cytokine mentions to obtain a more comprehensive characterization of the LSD cytokines. On the other hand, cytokine mentions not linked to any disease-related concept were not considered to avoid false-positive results. For example, if a sentence mentioned a cytokine related to an immune cell but did not specify any disease-related concept, this sentence was not included among the results because it could be potentially referred to another condition. We are aware that this approach can lower the recall rate and hamper the identification of some cytokines truly associated with LSDs but at the same time it increases the precision. Future extensions of this analysis could consider the extraction of relations within paragraphs to increase the number of cytokine-disease relations diving into “discourse parsing” and more research into coreference and anaphora resolution (Soricut and Marcu, 2003; Sukthanker et al., 2020). Another factor that can limit the power of the text-mining analysis is the unavailability of the entire document for many disease-relevant articles (Figure 1). Indeed, a recent study showed that text mining of full-text articles to identify protein–protein, disease-gene, and protein subcellular associations outperforms the analysis using abstracts only (Westergaard et al., 2018).

To extend the analysis we performed, other players of the inflammatory response, such as immune cells and molecules belonging to the complement system, could be included in the analysis. Indeed, a dysregulation of the complement pathway has been described in GD (Pandey et al., 2017, 2018) and its investigation in a systems biology framework could provide hints on the interplay with other inflammatory players. The integration of external data sources, such as gene expression data of immune cells, TF-cytokine gene interactions, and LR interactions allowed us to gain insights into the inflammatory signaling network. Indeed, cytokines are molecular effectors involved in cell-cell signaling (Armingol et al., 2020), and their production is regulated at the transcriptional level by combinations of TFs (Pro et al., 2018; Santoso et al., 2020). In this study, to evaluate the cell specificity and build the GRN, we relied on gene expression data of blood immune cells from the blood of healthy donors. Our literature-derived list of GD cytokines is significantly enriched in monocyte-specific cytokines. This finding is in agreement with the hallmark of GD pathophysiology, i.e., the accumulation of GL1 in the cells of the macrophage-monocyte system. Indeed, macrophages are the mediators of the removal of erythrocytes and leukocytes, which contain large amount of GL1 whose accumulation leads to clinical manifestations such as splenomegaly and hepatomegaly (Stirnemann et al., 2017). The significance of the TF-cytokine interactions in the GD monocyte network was tested by computing the gene expression correlation. This analysis allowed us to identify three TFs, namely STAT2, JUN, and NR4A2, that could be further investigated in the context of GD. The HPA Blood Atlas dataset used to perform the correlation analysis, however, includes only six samples and thus the analysis had a limited statistical power. The availability of disease-specific immune cell transcriptomics datasets, for example derived from single-cell sequencing experiments, would allow investigating more precisely the inflammatory signaling characterizing these diseases and to consider additional disease-specific immune cell types.

Our results can be used as a basis to further investigate the interplay between lipid accumulation and inflammation. To support drug discovery and development for LSDs, we recently developed quantitative systems pharmacology (QSP) models for GD type I and for ASMD, and a QSP platform that also includes FD is under development (Kaddi C. et al., 2018; Kaddi C. D. et al., 2018; Abrams et al., 2020). QSP models are mathematical tools that allow studying *in silico* the perturbations exerted by drugs on a biological system and test hypotheses on their mechanism of action. Literature mining can be effectively incorporated in the multistep process that leads to model development, becoming particularly useful for the definition of the model scheme and facilitating the identification of key biological processed to represent, along with data sources and parameter constraints needed for the model development (Azer et al., 2021).

## Data Availability Statement

Publicly available datasets were analyzed in this study. The data can be found here: http://tcm.zju.edu.cn/celltalkdb/, http://www.proteinatlas.org, https://cytreg.bu.edu/search.html. Scientific articles processed with NLP methods were retrieved from PubMed and PubMed Central.

## Author Contributions

SP, CK, KA, and RL conceived the study. SP and RL designed the analyses, curated the data, and wrote the original draft. SP, RL, PB, and DT performed the analyses. DT, AR, and RL developed the text-mining pipeline. ED and SN-Z supervised the project. ED managed the project and provided resources. SP, DT, PB, AR, CK, KA, ED, SN-Z, and RL discussed the results and reviewed the draft. All authors contributed to the article and approved the submitted version.

## Conflict of Interest

CK and SN-Z are current employees of Sanofi and may hold shares and/or stock options in the company. KA was employee of Sanofi while the initial phase of this study was conducted. SP, RL, DT, PB, and ED were contracted by Sanofi while the initial phase of this study was conducted. The remaining author declares that the research was conducted in the absence of any commercial or financial relationships that could be construed as a potential conflict of interest.

## Publisher’s Note

All claims expressed in this article are solely those of the authors and do not necessarily represent those of their affiliated organizations, or those of the publisher, the editors and the reviewers. Any product that may be evaluated in this article, or claim that may be made by its manufacturer, is not guaranteed or endorsed by the publisher.

## Footnotes

1
https://lucene.apache.org/solr/

2
https://www.omim.org/

3
https://www.orpha.net/

4
https://icd.who.int/

5
https://www.ncbi.nlm.nih.gov/mesh/

6
https://disease-ontology.org/

7
https://www.ncbi.nlm.nih.gov/gene

8
https://www.uniprot.org/

9
https://medlineplus.gov/

10
https://pubchem.ncbi.nlm.nih.gov/

11
https://www.ebi.ac.uk/chebi/

12
http://www.proteinatlas.org

13
https://cytoscape.org/

14
https://igraph.org

15
http://ncbi.nlm.nih.gov/geo/

16
https://www.ebi.ac.uk/arrayexpress/

17
https://www.omicsdi.org/

## Supplementary Material

Supplementary Figure 1 Linguistic structure of the sentences with cytokine-disease associations in the FD literature.

Supplementary Figure 2 Linguistic structure of the sentences with cytokine-disease associations in the ASMD literature.

Supplementary Table 1 Selected GD and FD transcriptomic and proteomic datasets.

Supplementary Table 2 Selected sentences from GD literature.

Supplementary Table 3 Selected sentences from FD literature.

Supplementary Table 4 Selected sentences from ASMD literature.

Supplementary Table 5 Results of text-mining analysis of TF search in the GD literature.

Supplementary Table 6 Significant results of TF-cytokine pairs correlation analysis.
