# Supplementary material for: Reconstruction of the Cytokine Signaling in Lysosomal Storage Diseases by Literature Mining and Network Analysis
Source: Front Cell Dev Biol. 2021 Aug 20;9:703489. doi: 10.3389/fcell.2021.703489 (PMC8417786; doi:10.3389/fcell.2021.703489)
Supplement: Supplementary Figure 1 — Linguistic structure of the sentences with cytokine-disease associations in the FD literature. [file Data_Sheet_1.pdf]

# *Supplementary Material*

## Supplementary Tables

**Supplementary Table 5.** Results of text-mining analysis of TF search in the GD literature

| Transcription Factors | PMIDS |
|---|---|
| DDIT3 | 24020503, 23034917, 30633777, 27856178 |
| JUN | 9272862 |
| IRF7 | 32831144, 27175482 |
| NR4A2 | 29198828 |
| STAT1 | 29317695 |
| STAT2 | 27175482 |
| VDR | 12439326, 19784695, 20419464, 20920864, 22388998, 23510066, 25978039, 30498352, 31791361 |
| XBP1 | 24020503, 31464647, 23034917, 19193629 |

**Supplementary Table 6.** Significant results of TF-cytokine pairs correlation analysis

| TF | cytokine | R | cor_p_val |
|---|---|---|---|
| HMGA1 | IL10 | -0.84586944 | 0.03380356 |
| HSF1 | CXCL2 | -0.92677261 | 0.00784704 |
| IRF1 | CCL2 | -0.83984713 | 0.03641954 |
| JUN | CCL2 | -0.89401437 | 0.01625417 |
| NFKB1 | CXCL9 | 0.89823323 | 0.01500774 |
| NFYC | CXCL9 | 0.91594223 | 0.0103016 |
| NR4A2 | TNF | 0.89937303 | 0.01467922 |
| SP1 | TNF | 0.8836823 | 0.01950783 |
| SPI1 | IL10 | -0.84443802 | 0.03441703 |
| STAT2 | CCL2 | -0.82553538 | 0.04300169 |
| STAT5A | IL10 | -0.86290337 | 0.02690483 |
| STAT6 | CCL2 | -0.83971423 | 0.0364783 |
| ELK4 | CCL2 | -0.84989588 | 0.03210585 |
| MAFF | IL10 | -0.97360467 | 0.00103587 |
| MAFG | IL10 | 0.81641087 | 0.04746352 |
| ZDHHC17 | CCL1 | -0.89694999 | 0.0153818 |
| ZFP90 | CCL1 | 0.85600512 | 0.02960896 |
| ZNF621 | CCL1 | -0.95706106 | 0.00272604 |
| ZNF710 | IL10 | -0.83399893 | 0.03904734 |
| ZNF710 | CXCL9 | 0.82047334 | 0.04545167 |

## Supplementary Figures

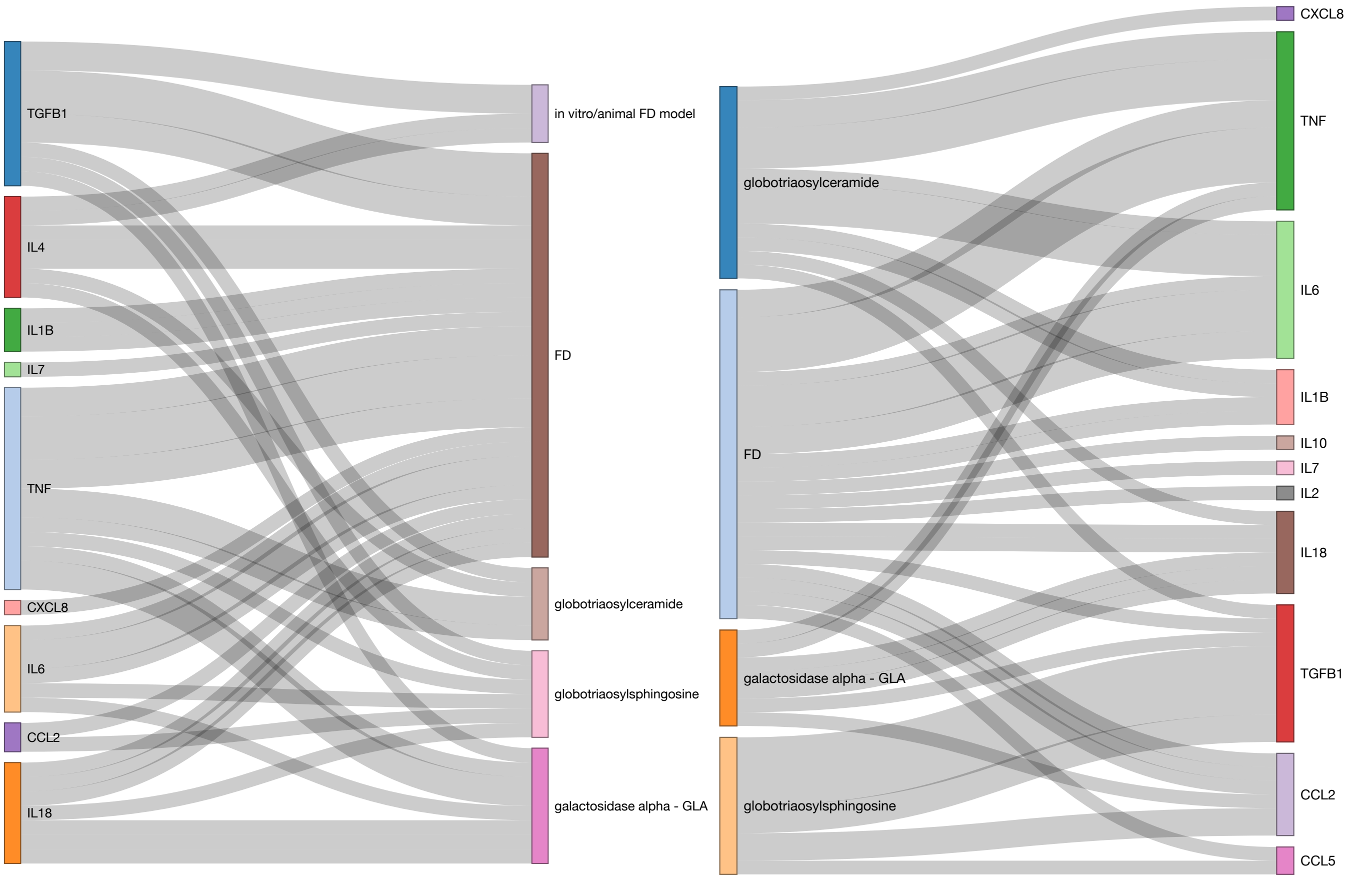


**Supplementary Figure 1.** Linguistic structure of the sentences with cytokine-disease associations in the FD literature

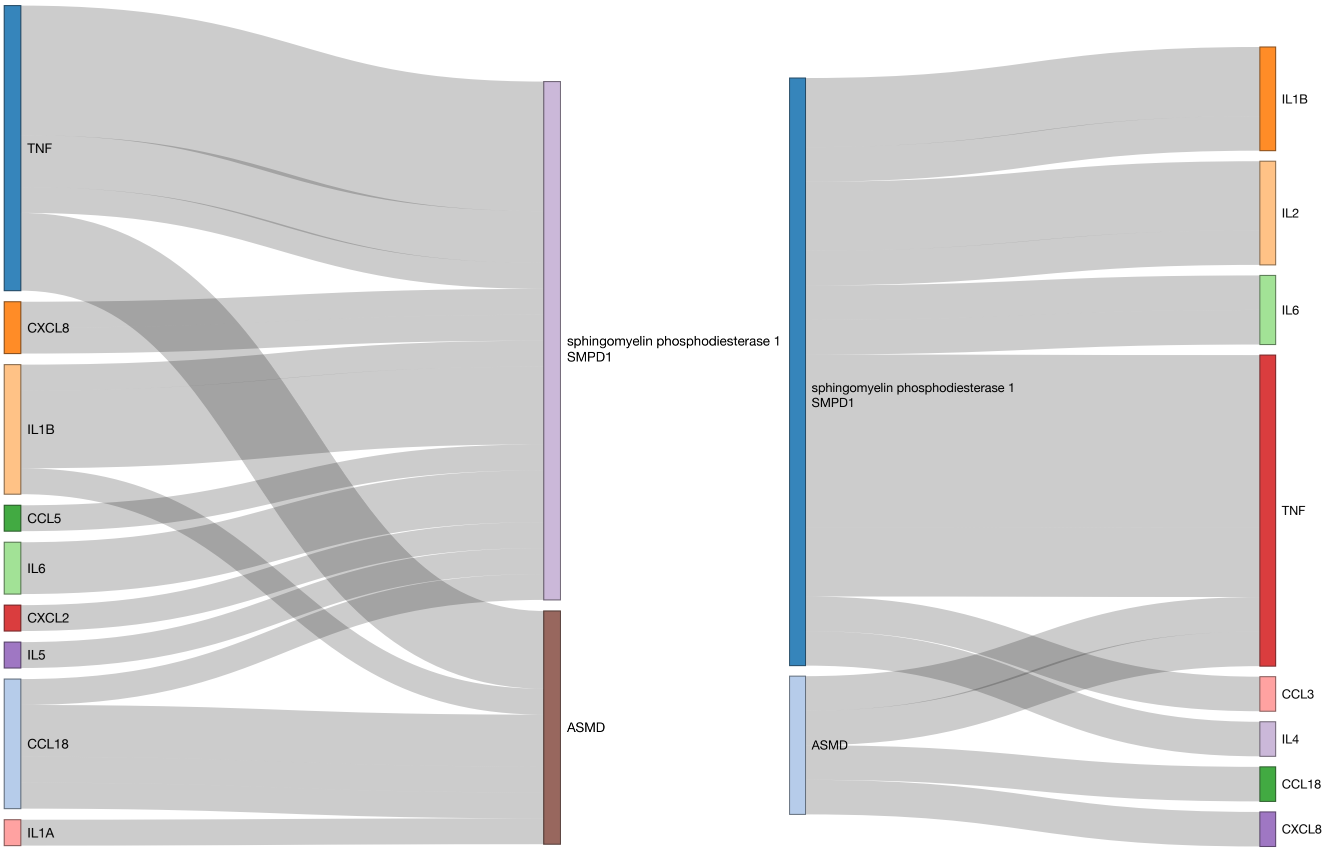


**Supplementary Figure 2.** Linguistic structure of the sentences with cytokine-disease associations in the ASMD literature
